# Source Attribution Study of Sporadic **Salmonella** Derby Cases in France

**DOI:** 10.3389/fmicb.2020.00889

**Published:** 2020-05-14

**Authors:** Yann Sévellec, Sophie A. Granier, Simon Le Hello, François-Xavier Weill, Laurent Guillier, Michel-Yves Mistou, Sabrina Cadel-Six

**Affiliations:** ^1^Laboratoire de Sécurité des Aliments, Agence Nationale de Sécurité Sanitaire de l’Alimentation, de l’Environnement et du Travail, Université PARIS-EST, Maisons-Alfort, France; ^2^Laboratoire de Fougères, Agence Nationale de Sécurité Sanitaire de l’Alimentation, de l’Environnement et du Travail, Fougères, France; ^3^Unité des Bactéries Pathogènes Entériques, Centre National de Référence des Escherichia coli, Shigella et Salmonella, Institut Pasteur, Paris, France

**Keywords:** *Salmonella* Derby, source attribution study, SNP analysis, human infection, antimicrobial resistance

## Abstract

*Salmonella enterica* subsp. *enterica* serovar Derby is one of the most frequent causes of gastroenteritis in humans. In Europe, this pathogen is one of the top five most commonly reported serovars in human cases. In France, *S.* Derby has been among the ten most frequently isolated serovars in humans since the year 2000. The main animal hosts of this serovar are pigs and poultry, and white meat is the main source of human contamination. We have previously shown that this serovar is polyphyletic and that three distinct genetic lineages of *S.* Derby cohabit in France. Two of them are associated with pork and one with poultry. In this study, we conducted a source attribution study based on single nucleotide polymorphism analysis of a large collection of 440 *S.* Derby human and non-human isolates collected in 2014–2015, to determine the contribution of each lineage to human contamination. In France, the two lineages associated with pork strains, and corresponding to the multilocus sequence typing (MLST) profiles ST39-ST40 and ST682 were responsible for 94% of human contaminations. Interestingly, the ST40 profile is responsible for the majority of human cases (71%). An analysis of epidemiologic data and the structure of the pork sector in France allowed us to explain the spread and the sporadic pattern of human cases that occurred in the studied period.

## Introduction

Non-typhoid *Salmonella enterica* subsp. *enterica* is a major cause of food illness causing self-limiting gastroenteritis, with 550 million people becoming ill each year, including 220 million children under 5 years of age (WHO, 2018). Salmonellosis is usually characterized by acute onset of fever, abdominal pain, diarrhea, nausea, and sometimes vomiting. The onset of symptoms occurs 6–72 h (usually 12–36 h) after ingestion of *Salmonella*, and the illness lasts 2–7 days (WHO, 2018). Symptoms of salmonellosis are relatively mild and in most cases, patients recover without specific treatment. However, in some cases, particularly in children and elderly patients, the infection can become severe and life-threatening (Feasey et al., 2012; Ao et al., 2015). In these cases, antibiotic treatment is recommended (Ruiz et al., 2004).

*Salmonella enterica* subsp. *enterica* serovar Derby (*S.* Derby) is one of the serovars that mainly affects at-risk populations, such as children and the elderly (Rubbo, 1948; Sanders et al., 1963; Simon et al., 2017). In 1946, *S.* Derby caused an epidemic in Australia, affecting 68 infants and young children and led to 10 fatal outcomes (Mushin, 1948). In 1963, an outbreak linked to contaminated eggs provoked 822 cases in 53 hospitals in the United States (Sanders et al., 1963). More recently, between the end of 2013 and the beginning of 2014, *S.* Derby was responsible for an outbreak linked to contaminated pork involving 145 patients, mostly elderly people in Berlin and the Brandenburg region in Germany (Simon et al., 2017). However, beyond these well-documented foodborne outbreaks, *S.* Derby is most often associated with sporadic human cases that cannot be attributed to any food source. In China, *S.* Derby is the third most commonly reported serovar in clinical cases (Ran et al., 2011), and the most frequent serovar reported in infants and toddlers (Cui et al., 2009). In Africa, few data are available about the diversity of this serovar, but it was reported in Ghana as the third most commonly isolated serovar in humans (Andoh et al., 2017). In the United States, since 2005, *S.* Derby has caused about 120 confirmed cases each year (CDC, 2017). In Europe, *S.* Derby is the 5th most frequently isolated serovar in humans, with 612 confirmed cases in 2017 (ECDC, 2018). In France, since 2000, this serovar has ranked between the 5th and 8th position (*n* = 113–178 clinical isolates) among human isolates (Weill and Le Hello, 2014; Weill et al., 2018).

The main reservoirs of this pathogen are pigs and poultry worldwide. In a study organized by the European Food Safety Agency in 2008, this pathogen was found to contaminate 28.5% of European swine livestock (EFSA, 2008), and since 2015, it has been recorded as predominant in turkey flocks (EFSA, 2015, 2016, 2017; Sévellec et al., 2018b). In Europe, in 2015, *S.* Derby accounted for 22.9% of isolates from pork sector, followed by *S*. 4,[5],12:i:- (i.e., the monophasic variant of *S.* Typhimurium) (22.3%) and *S.* Typhimurium (20.6%) (EFSA, 2016). *S*. Derby is the 4th most frequently isolated serovar in the non-human sectors in the United States (Schmidt et al., 2012), and the main serovar isolated from slaughter pigs in China (Cai et al., 2016). Among the Derby strain collected in Europe, 66, 21, and 11% were isolated in 2016 form pork, turkey, and broiler, respectively (EFSA, 2017). In France, ANSES *Salmonella* Network data ranked *S.* Derby in 4th position of the most frequently isolated serovar, right after Typhimurium, its monophasic variant and Enteritidis (Leclerc et al., 2016). In 2014–2015, *S*. Derby was mainly isolated from pork and poultry meat: out of a total of 414 isolates 44% (183/414) and 30% (126/414) were originated from pork and poultry meat, respectively. A former source attribution study identified meat products as the source of more than 50% of the salmonellosis in France (David et al., 2011). Moreover, nationwide surveillance program established *S*. Derby prevalence in pork, poultry and beef carcasses to be >10%, >12%, and <1%, respectively (DGAL, 2014, 2015). Altogether, *S*. Derby might represent a significant threat to human health, and the contribution of pork and poultry sectors deserve to be further investigated.

Our previous genomic analysis conducted on a collection of 141 non-human *S.* Derby strains isolated between 2014 and 2015, showed three main genomic lineages in France, two associated with the pork sector (ST39-40 and ST682), and one with poultry (ST71) (Sévellec et al., 2018b, 2019). The high prevalence of *S.* Derby in these two sectors, which account for two-thirds of meat consumption in France, highlights the need to clarify the source of sporadic human cases of *S*. Derby. Except for a retrospective investigation of the *S*. Derby German outbreak occurred in 2014 (Simon et al., 2017), no whole genome sequencing (WGS) investigation of *S*. Derby serovar has been conducted so far in the European Union. Previous attempts to subtype *S.* Derby using MLST and pulsed field gel electrophoresis (PFGE) typing and attribute non-human strains to human cases were either limited in scope and considered only the pork sector (Hauser et al., 2011; Kerouanton et al., 2013; Zheng et al., 2017), or lacked a representative collection of strains to encompass the genetic diversity of *S.* Derby (Hayward et al., 2016).

We carried out a source attribution study based on core-genome analysis of 440 *S*. Derby strains, 299 of which correspond to all sporadic human cases associated with *S.* Derby that occurred in France in 2014 and 2015. The results enabled us to identify the *S*. Derby clone responsible for most human cases in France and linked that contamination event to food samples. In addition, the accessory genome was also taken into account through analysis of the antimicrobial resistance genotype.

## Materials and Methods

### Construction of the Genomic Dataset

The whole collection of 141 non-human *S.* Derby strains previously published in 2018 (Sévellec et al., 2018a) was used in this study. This collection included food strains from pork (*n* = 85) and poultry (*n* = 56) sectors. Strains were isolated from raw material (carcass, offal, and fresh meat) or processed meat products (sausage) sampled at slaughterhouses, processing plants and retail markets. *S*. Derby strains from beef were excluded due to their very low frequency in the cattle sector (<1%) (Leclerc et al., 2016). Strains isolated from ovine meat were also excluded due to the low consumption of sheep and goat meat in France (<3%) (Menard et al., 2015).

The 299 human strains corresponding to all the clinical cases that occurred in metropolitan France in 2014 and 2015 were collected by the National Reference Center (NRC) (*Institut Pasteur*). These strains were identified as serovar Derby by glass slide agglutination, according to the White–Kauffmann–Le Minor scheme (Grimont and Weill, 2007), and their genomes were extracted and sequenced as previously described (Ung et al., 2019). Therefore, the total French dataset used for the source attribution and comparative genome studies comprised 440 genomes.

Genome accession numbers, relevant epidemiologic data and sequence quality metrics for all the genomes used in this study are reported in Supplementary Table S1.

### Multilocus Sequence Typing (MLST)

All genomes were characterized by *in silico* MLST using seven housekeeping genes *aroC*, *dnaN*, *hemD*, *hisD*, *purE*, *sucA*, and *thrA* (Achtman et al., 2012). The seven housekeeping gene sequences for each strain were uploaded to the MLST service of the Center for Genomic Epidemiology^1^ (CGE), which allowed us to determine the sequence type (ST) directly from the read files.

### Single Nucleotide Polymorphism (SNP) and Phylogenetic Analyses

The SNP analysis of the 440 strains was conducted using the iVARCall workflow (Felten et al., 2017). The 2014LSAL02547 *S.* Derby reference sequence (NCBI accession number CP029486) (Sévellec et al., 2018a) was used to generate the VCF files. Phylogenetic analyses were computed using RAXML software (Stamatakis, 2014). The phylogenetic trees were constructed under the maximum likelihood criterion using the GTR-gamma model of nucleotide evolution. The phylogenetic analyses were based on the pseudogenome obtained using GATK (McKenna et al., 2010).

For each lineage, a subtree was constructed as described above. The genome of strain 2014LSAL02547 (NCBI no. CP029486) (Sévellec et al., 2018a) was used as a reference for the ST40 and ST39 analysis (average breath coverage of 99.9 and 96.4%, respectively) and the genome of strain 2014LSAL01779 (NCBI no. CP026609) (Sévellec et al., 2019) was used as a reference for the ST71 analysis (average breath coverage of 99.9%). In the absence of a complete genome for ST682, *S.* Typhimurium LT2 (NCBI NC_003197.1) was used as a reference (average breath coverage of 91.6%). Phylogenetic trees were constructed using iqtree V 1.6.9 (Schmidt et al., 2014) with comparable results with RaXml.

Statistical confidence levels for all topologies were evaluated by the non-parametric bootstrap method (1,000 replicates). The phylogenetic data were visualized using interactive Tree Of Life (iTOL^2^) (Letunic and Bork, 2016).

### Statistical Analyses

The non-normality of the data (number of paired SNP differences) was assessed using a Shapiro test (Royston, 1995) on R, from the pairwise matrix obtained. The comparison between the paired SNP differences was tested by a Kolmogorov–Smirnov test (KS-test) (Huang et al., 2016), provided the variance of the distribution by paired SNP is proven significantly unequal by the Fisher test (Markowski and Markowski, 1990).

### Identification of Acquired Resistance Genes

The collection of genomes in the dataset was analyzed by the ResFinder 2.1 application (Zankari et al., 2012) on the CGE server, as described previously (Sévellec et al., 2018b). The localization of resistance genes inside the assembled genomes was tested by running a blast on Bionumerics V. 7.6.1. The structure of the *S*. Derby class 1 integron (NCBI: HG314953.2) was obtained from the whole database (Moura et al., 2009), and the presence of this integron was tested against our collection using the BLAST tool.

## Results

### Genetic Diversity and Source Attribution Study of the Collection

**FIGURE 1:**
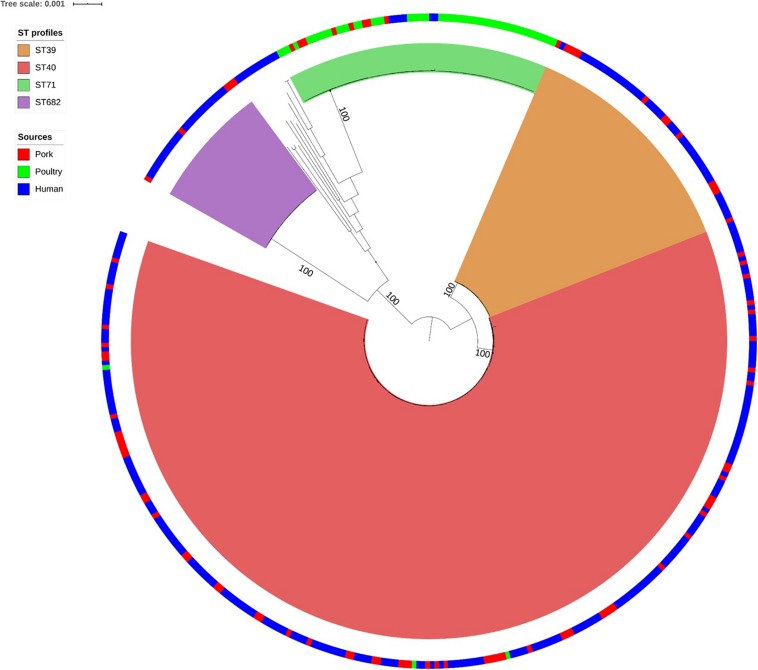
Phylogenetic tree of the 440 French *Salmonella* Derby human and non-human isolates. Maximum likelihood tree based on 141 food isolates associated with pork (red in the ring), poultry (green in the ring), and 299 human isolates (blue in the ring). Rooting was performed based on an outgroup comprising two *S*. Typhimurium and one *S*. Newport genome. Bootstraps supports the four genetic lineages reported herein are 100%.

As shown in Figure 1, the SNP analysis clustered the human and non-human *S*. Derby genomes into four main clusters, corresponding to the MLST groups ST39, ST40, ST71, and ST682. Therefore these ST profile names were used to label the clusters identified by SNP analysis. We observed that 94% of the human strains from France clustered into the three genetic groups associated with pork: ST39 (44/299), ST40 (211/299), and ST682 (25/299); while only 6 human strains (2%) belonged to the poultry-related group: ST71. Only 12 human strains (4%) did not belong to one of these four groups and belong to rare ST profiles that have already been described (ST683, ST3135, and ST5420: 3 strains in total associated with human cases in Europe) or to new ST profiles (ST5423, ST5424, ST5426, ST5428, ST5433, ST5434, and ST5437: 9 strains in total) (Supplementary Table S1). Finally, the strains 201501530 and 201503142 presented incomplete ST profiles but were presumably related to ST40 and ST682, respectively.

Within each of the four clusters, the core-genome genetic distance between the strains was less than 270 SNPs. The largest genetic distance was observed for ST39 (average of 177 ± 93 SNPs) (Table 1), the smallest for ST40 (39 ± 19 SNPs).

**TABLE 1:** Genetic distance (based on average pairwise SNPs) between each cluster of strains.

**Clusters**	**ST39**	**ST40**	**ST71**	**ST682**
**ST39**	177 (±93)	5,175(±42)	34,436(±24)	37,466(±23)
**ST40**		39(±19)	34,120(±49)	37,589(±10)
**ST71**			34(±60)	38,992(±20)
**ST682**				63(±22)

Genomes belonging to ST39 and ST40 are related to an average of 5,175 SNPs and a standard deviation (SD) of 42 SNPs. The strains belonging to ST71 were distant from ST40 by 34,120 SNPs and an SD of 49 SNPs. ST682 was the most genetically distant from ST39, ST40 and ST71 with an average of 37,841 ± 10 SNPs (Table 1).

Considering the epidemiological information available for both human and non-human strains, there was no evidence of a relationship between genetic relatedness and geographical localization within the different lineages. Closely related strains could have very different geographic origins and *vice versa*.

The four lineages of *S.* Derby showed a very wide geographic distribution, clustering together strains from Pays-de-la-Loire, Bourgogne, Languedoc-Roussillon and Centre, regions several hundred kilometers apart. Strains isolated in Pays-de-la-Loire displayed considerable diversity in terms of ST profiles; these strains belonged to the four different lineages. Consistent with the distribution of the pork and poultry food production chains in France, ST40 was geographically more widespread than ST71. As previously described by Sévellec et al. (2018b), we can clearly associate three genetic groups with the pork sector (ST39, ST40, and ST682), and one with the poultry sector (ST71).

#### Cluster ST40

**FIGURE 2:**
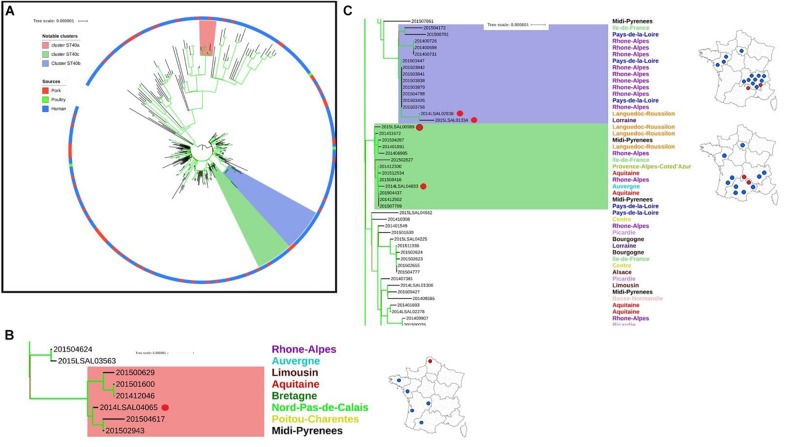
Phylogenetic tree of the French *Salmonella* Derby isolates belonging to ST40. **(A)** Complete phylogenetic tree: two clades can be identified within Cluster ST40. The ring color reports the source of the strain: pork (red), poultry (green), and human (blue). Bootstraps are presented by a color range on the nodes, varying from green (100% of the bootstraps supports the given node) to red (4% of the bootstraps supports the node). Images **(B,C)** illustrate red, purple, and green groups underlined in the image **(A)**. **(B)** Red group: this group belongs to Cluster ST40 clade 1. It includes one food strain isolated from Nord-Pas-de-Calais (red dot) with 5 human strains spread in the west of France. **(C)** Purple and green groups: these groups belong to Cluster ST40 clade 2; rough geographic localization of the human strains and related non-human strains (red dots) are provided on the maps of France.

The ST40 population can be divided into two distinct clades. Clade 1 presents core-genome diversity of 62 ± 19 SNPs, and Clade 2 presents diversity of 16 ± 5 SNPs (Figure 2A).

In Clade 1, five human strains (201500629, 201501600, 201412046, 201504617, and 201502943) isolated from patients from across the west of the French territory are related to food strain 2014LSAL04065 isolated in the north of France from pepper sausage (Figure 2B). These strains present 6 ± 4 SNP differences in their core-genomes and share the same profile of antimicrobial resistance genes. Analysis of the resistome confirmed this finding (see Supplementary Figure S1).

In Clade 2, two groups of related strains were identified. Food strains 2014LSAL02838 and 2015LSAL01334 are likely associated with 13 human cases mainly reported in the Rhône-Alpes region (9/13), with 6 ± 4 SNP differences in their core-genomes (Figure 2C, purple group). Food strains 2014LSAL04833 and 2015LSAL00989 are related to 11 human cases, with 3 ± 2 SNP differences in their core-genomes (Figure 2C, green group). Antimicrobial resistance encountered in ST40 is presented in Supplementary Figure S1.

#### Cluster ST71

**FIGURE 3:**
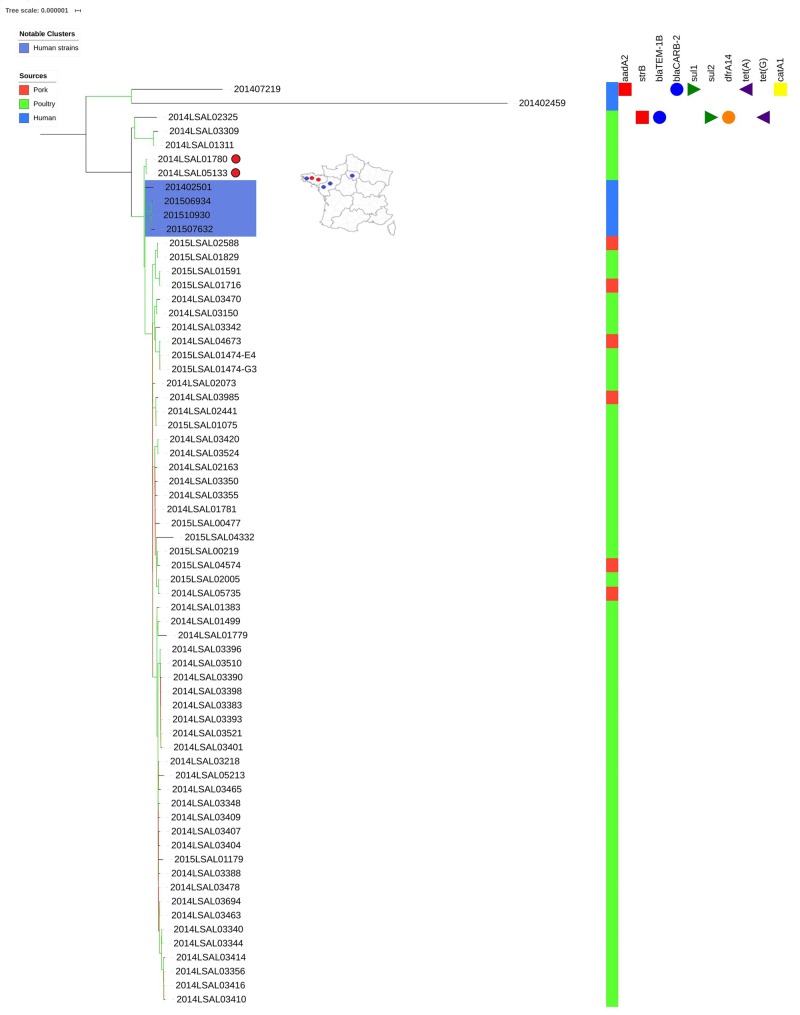
Phylogenetic tree of the French *Salmonella* Derby isolates belonging to ST71. The colored zone alongside the tree represents the source of the strain: pork (red), poultry (green), and human (blue). The predicted antibiotic resistance genes are indicated on the right-hand side. Red cubes correspond to the resistance genes to aminoglycoside antibiotics, blue circles to beta-lactams, green triangles to sulfonamides, orange circles to trimethoprim, purple triangles to tetracycline and yellow squares to phenicols. Bootstraps are presented by a color range directly on the nodes, varying from green (100% of the bootstraps supports the given node) to red (4% of the bootstraps supports the node). For the human strains (blue color range) and related non-human strains (red dots), the rough geographic localization of the human strains (blue dots) and related non-human strains (red dots) are provided on the map of France.

As demonstrated in our previous study (Sévellec et al., 2019) within the poultry sector, we were able to group separately two human strains (201402459 and 201507219) from the other strains. Interestingly, the strain 201402459 was associated with travel to Thailand. These two human strains differ from the others by an average of 457 SNPs and 199 SNPs, respectively. With the exception of these two strains, all the others show little genetic diversity (25 ± 9 SNPs on average). Within these related strains, the only 4 human clinical strains were clustered together and distant by 8 ± 6 SNPs on average (Figure 3, strains in the blue box). These human strains were closely related to two food strains, strain 2014LSAL01780 isolated in Brittany from a *Gallus gallus* carcass at the slaughtering stage at the end of April 2014, and strain 2014LSAL05133 isolated in Brittany from a turkey carcass at the slaughtering stage at the beginning of November 2014, both distant by 11 ± 2 SNPs from the clinical strains. In the cluster ST71, the only 6 strains isolated from pork (Supplementary Table S1 and Figure 3) spread inside the mains cluster along with 45 poultry strains, with 14 ± 2 SNP on average. However, these strains isolated from pork couldn’t be linked to any human cases. Figure 3 illustrates the genetic diversity of ST71 in France.

#### Cluster ST39

**FIGURE 4:**
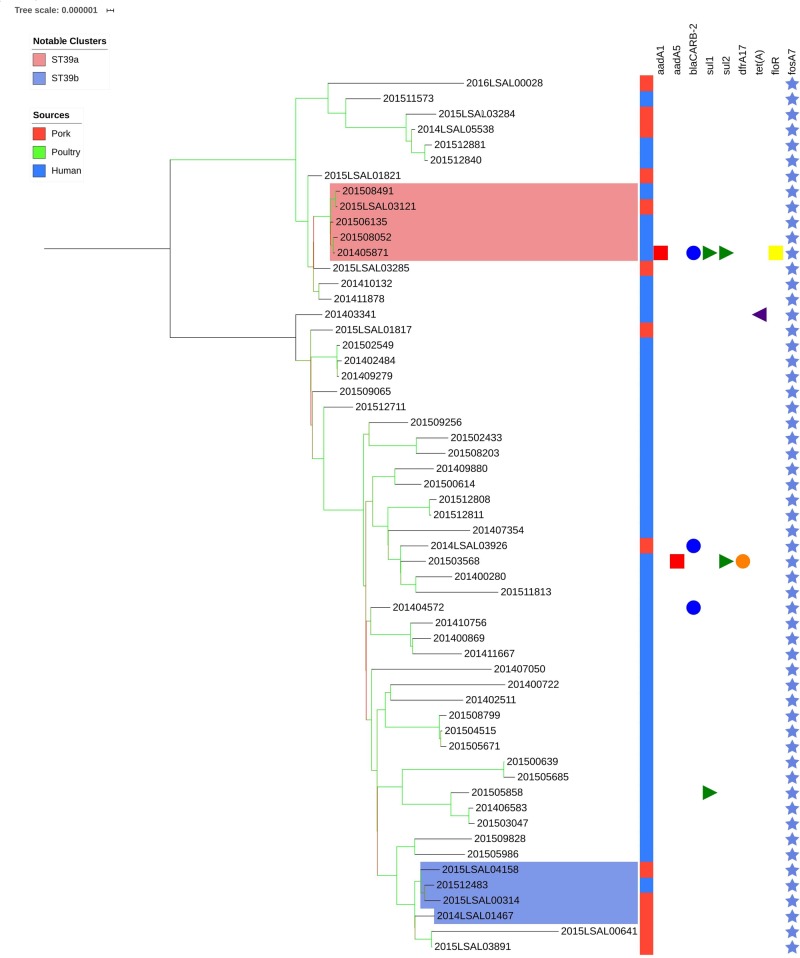
Phylogenetic tree of the French *Salmonella* Derby isolates belonging to ST39. The colored zone alongside the tree represents the source of the strain: pork (red), poultry (green), and human (blue). The predicted antibiotic resistance genes are represented on the right-hand side. Genes conferring resistance to the main antibiotic classes are presented in different colors: red squares for aminoglycosides, blue circles for beta-lactams, green triangles for sulfonamides, orange circles for trimethoprim, purple triangles for tetracyclines, yellow squares for phenicols, and light blue stars for fosfomycins. Bootstraps are presented by a color range on each node from green (100% of the bootstraps supports the given node) to red (4% of the bootstraps supports the node).

Figure 4 illustrates the genetic diversity of ST39 in France. ST39 presented broader genetic diversity than the other lineages as the strains were distant of 177 ± 93 SNPs. ST39 could be divided into two clades distant by an average of 294 ± 19 SNPs. Strains in Clade 1 were distant by an average of 74 ± 48 SNPs from each other. Strains in Clade 2 were distant by 107 ± 33 SNPs on average. Within Clade 1, the food strain 2015LSAL03121 was related to 4 clinical strains (201508491, 201506135, 201508052, and 201405871) with an average of 5 ± 4 SNPs in their core-genomes (Figure 4, red group). In Clade 2, the clinical strain 201512483 could be associated with four food strains isolated from Auvergne (2014LSAL01467, 2015LSAL03891 and 2015LSAL04158) and Languedoc-Roussillon (2015LSAL00314) (26 ± 13 SNPs on average) (Figure 4, blue group).

#### Cluster ST682

**FIGURE 5:**
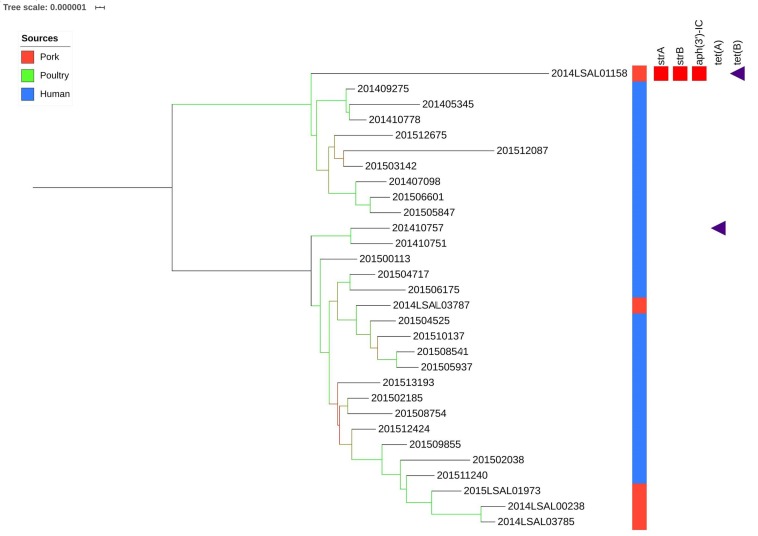
Phylogenetic tree of the French *Salmonella* Derby isolates belonging to ST682. The colored zone alongside the tree represents the source of the strain: pork (red), poultry (green), and human (blue). The predicted antibiotic resistance genes are indicated on the right hand side. Red cubes correspond to aminoglycosides resistance genes and purple triangle to tetracycline resistance genes. Bootstraps are presented by a color range on each node, varying from green (100% of the bootstraps supports the given node) to red (4% of the bootstraps supports the node).

Figure 5 illustrates the genetic diversity of ST682 in France. ST682 was homogenous and presented an average distance between the strains of 63 ± 22 SNPs. No significant association could be established between the food strains (*n* = 5) and human strains (*n* = 25) belonging to this clade.

### Antimicrobial Resistance

The antimicrobial resistance gene profiles were obtained for the 440 genomes (Supplementary Table S2). We observed that 97% (62/64) and 93% (28/30) of strains belonging to ST71 and ST682, respectively, were devoid of any known antimicrobial resistance genes, while this was only the case for 17% of the ST40 strains (48/275). All ST39 carried the *fosA7* gene involved in resistance to fosfomycin (Rehman et al., 2017) and a close homolog (97% identity) was present in 99.3% of the ST40 isolates.

The simultaneous carriage of the *aadA2*, *sul1* and *tetA* genes corresponding to resistance to aminoglycosides, sulfonamides and tetracyclines (STR-SSS-TET profile) was common among ST40 isolates: 62% of human strains and 82% of non-human strains. Twenty strains presented resistance for 4 antibiotic classes or more: 16, 2 and 2 strains from ST40, ST71, and ST39, respectively.

## Discussion

In this study, the genomes of *S.* Derby from human and non-human (food and environmental) strains were analyzed by SNP phylogenetic analysis to investigate the reservoirs responsible for sporadic human cases in France during the years 2014 and 2015. This constitutes the first WGS-based source attribution study for *Salmonella* in France. WGS-based source attribution is only possible when networks of laboratories that share genomic (and epidemiologic) data, work together toward harmonizing methods, inputs, and outputs (Adam and Brülisauer, 2010; Mughini-Gras et al., 2018).

Of the 299 human strains isolated in 2014–2015, 94% belonged to STs associated with the pork sector (ST39, ST40, and ST682) (Sévellec et al., 2018b). Only 2% of clinical strains belonged to the poultry specific ST71 cluster. In case of a higher diversity of sources in STs or a higher difference in relative meat consumption, it would have led us to apply frequency-matching source attribution models. Considering the strong association observed between STs and sources, the use of sophisticated source attribution models did not appear relevant here (Mughini-Gras et al., 2019).

Interestingly, the *S.* Derby genomes of human origin displayed higher genomic diversity, with 11 ST profiles that were not present in our collection of food isolates. They were found only among these isolates and were genetically distant from the clades isolated from food. The origin of contamination by these rare STs could be either unreported history of travel or other animal sources that were not investigated in our datasets, such as bovine or ovine reservoirs or imported food products. Further analysis would be needed to confirm this hypothesis.

The *S.* Derby clinical isolates from ST40 included in Clades 1 and 2 represent 21 and 50% of the studied clinical collection, respectively, making ST40 isolates responsible for 71% of the human cases identified in France in the 2014–2015 period. Clade 1 corresponds to more distantly related strains with 62 SNPs on average vs. 16 SNPs in Clade 2. Clade 2, the main clade responsible for human infection in France, presents broad genetic homogeneity. The strains belonging to Clade 2 were characterized by *S.* Derby genomic island-1 (SGI-1) (Beutlich et al., 2011; Sévellec et al., 2018b) with antibiotic resistance genes associated with the profile STR SSS TET and a mercuric resistance operon *mer*. The genetic homogeneity and the geographic dispersion of strains belonging to Clade 2 across the whole French territory illustrate the adaptive advantage of SGI-1 acquisition. In fact, since aminoglycosides, sulfonamides and tetracyclines are the most commonly used antibiotics in the pork sector (Méheust et al., 2016), this resistance profile might explain the success of this particular clade (Newell et al., 2010). This resistance profile was already reported in Spain in 2005 (Valdezate et al., 2005), in France in 2013 (Kerouanton et al., 2013), and is widespread in Europe in human and pork *S.* Derby strains (Bonardi, 2017). Moreover, resistance to heavy metals such as mercury has been demonstrated as a co-selection factor for antimicrobial resistance as this is often carried by the same transposons (Baker-Austin et al., 2006; Pal et al., 2015; Deng et al., 2017). The presence of resistance to heavy metals was already observed in Europe in the major clone of the monophasic variant of *Salmonella* Typhimurium (Petrovska et al., 2016).

In ST40 Clade 2, the use of a closely related reference genome (99.97% breath coverage on the alignment) allowed us to identify two groups of related strains associating food strains 2014LSAL02838 and 2015LSAL01334 with 13 human strains (6 ± 4 SNPs), and food strains 2014LSAL04833 and 2015LSAL00989 with 11 human strains (3 ± 2 SNPs) (Figure 2C).

In the first group of strains (purple group in Figure 2C), we identified two events of human contamination which occurred mainly in the Rhône-Alpes region: one occurred in January 2014 and the second in May–June 2015 with a few sporadic cases isolated in Pays-de-la-Loire (*n* = 3) and Ile-de-France (*n* = 1). The human isolates involved in the January 2014 first wave of contamination that occurred in the Rhône-Alpes region were genetically identical. Three of the cases that occurred in 2015 were identified in Belleville (Rhone) between the 9th and the 16th of May, suggesting the common source of contamination. The non-human strains associated with these two contamination events came from two different facilities in adjacent administrative departments (Lozère and Drôme). Strain 2014LSAL2838 was isolated in July 2014 from a pig carcass in Lozère, and strain 2015LSAL00989 from a pig head in March 2015 in Drôme. These isolates reveal the persistence of this *S.* Derby clone within the local pork production sector.

The group of isolates highlighted in green (Figure 2C) gathers 11 clinical strains isolated from across the French territory between August 2014 and December 2015. The two associated non-human strains, 2014LSAL04833 and 2015LSAL00989, were isolated in September 2014 and March 2015 from pork minced meat and a pig carcass in two bordering administrative departments, Cantal and Lozère, respectively. These results highlight the dispersion of a persistent virulent ST40 Derby clone along the local production chain. The French east-center and southwest pork production basin (Center, Auvergne-Rhône-Alpes, Midi-Pyrénées, and Languedoc-Roussillon regions including Lozère, Drôme and Cantal departments) is characterized by small slaughterhouses which support local production and covers 5.4% of national production (Supplementary Figure S2). In France, 75% of slaughtering is carried out in a few large slaughterhouses located in the north-west of the country that handle industrial transformation and provide retail sales nationwide.

Slaughterhouses represent a vulnerable spot for microbiological contamination, as described in many publications (Rostagno et al., 2003; Botteldoorn et al., 2004; Sofos, 2008; Fois et al., 2017). It is also known that *S.* Derby is persistent in swine population (Valdezate et al., 2005; EFSA, 2009); recurrent contamination by a dominant clone persistent in a breeding and holding facility is a strong possibility (Henry, 2014). This phenomenon of a continuous supply of a persistent clone via livestock is observed in other *Salmonella*, such as the monophasic variant of Typhimurium (Gosling et al., 2018). The collection developed for this study will constitute an asset to investigate the source of contamination across the food chain. Finally, because *Salmonella* is able to survive freezing processes (Chaves et al., 2011), another hypothesis could be that frozen meat stocks distributed over a longer period of time might help to explain the temporal dispersion of human cases linked to virulent clones.

Another example that shows the role of scattered pork product distribution in the dispersion of the virulent Derby clones is highlighted in Figure 2B. Food strain 2014LSAL04065, isolated from a dry sausage (*miche au poivre*) in the north of France, is closely related to 5 human cases dispersed in the west of France and isolated from November 2014 to April 2015. These strains are all characterized by a particular antibacterial resistance gene profile (STR SMX TET TMP) (Supplementary Figure S1). As already highlighted in a previous study (Sévellec et al., 2019), the analysis of the accessory genome (i.e., via the antimicrobial resistance gene profiles) is complementary to core-genome analysis, and is highly recommended when the objective is to establish links between food and human isolates within low-diversity clades.

Although ST40 is responsible for most human infections in France, ST39 and ST682 also participate with 15 and 7% of human cases, respectively.

Importantly, within the strains belonging to ST39 and ST682, a small number of human strains have been linked to food strains (Figures 4, 5). This can be explained by the number of ST39 and ST682 food strains in our dataset. The inclusion of more food strains from these two lineages and the use of a more accurate reference genome for the analysis of ST682 strains would probably allow a better understanding of the diversity of these lineages and the sources of human contamination. Nonetheless, two clusters group human and food strains within ST39 (cluster ST39a and ST39b; Figure 4).

Remarkably, most of the *S.* Derby genomes without any antimicrobial resistance genes identified in France belong to ST682 (28/30 genomes) and ST71 (62/64 genomes). The presence of a resistance gene for fosfomycin *fosA7* (Rehman et al., 2017) was detected in all genomes from ST39 and a 97% identity variant is present in all the genomes of ST40. These genes were not reported in previous studies (Sévellec et al., 2018b) as this gene was included only recently in the Resfinder database. This result demonstrates the importance of keeping track of the updates of public databases since some resistance genes may go unnoticed, depending on the database version. Resistance to fosfomycin was not tested in our antimicrobial susceptibility test, nor in previous publications to our knowledge. Further antimicrobial susceptibility tests should be performed to verify the functionality of the *fosA7* gene both in ST39 and in ST40.

Only 6 human cases were caused by strains from ST71. Two of these strains were linked to strains from Asia in a previous publication (Sévellec et al., 2019). Strain 201402459 had already been associated with travel to Thailand, but phylogenetic analysis made it possible to link strain 201407219 to strains from Asia in ST71. In the European cluster, all 4 human strains could be associated with 2 food strains isolated from chicken and turkey. Both strains were isolated from slaughterhouses in the Côte d’Armor administrative department between April and November 2014. Within the ST71 cluster, 6 strains were isolated from pork; nevertheless, no one human strain was associated with them. Interestingly, 5 of these strains were isolated at slaughterhouses and processing plants in the South/South-Est of France (Occitanie-Auvergne-Rhône-Alpes) and one strain in a retail market in Ile-de-France (Paris area). Zheng et al. (2017) also identified in 2017 in China some ST71 strains isolated from the pork sector but only at slaughterhouse and retail market levels. These results can suggest that those strains might have an environmental origin and might have contaminated the meat during or after slaughter. Finally, all these results suggest that ST71 strains are probably less adapted to human host than other *S*. Derby. In order to assert this hypothesis, we are currently carrying on an investigation of the virulence factors of the four different lineages (ST40, ST39, ST682, and ST71) and *in vivo* cellular tests are currently underway. In the meanwhile, investigation of accessory genomes could also enable to explore the host range differences observed using WGS analysis.

As the proportion of pork and poultry meat in the total meat consumption in France was 38% (IFIP, 2014) and 29% (Malpel et al., 2014), respectively, the difference in the contribution of the lineages of *S.* Derby specific to the pork and poultry sectors can be explained either by differences in handling and cooking habits for pork and poultry meat or by an impaired capacity of ST71 strains to cause illness in humans. Hayward et al. (2016) demonstrated significant differences in the adaptation to pork between the lineages of *S.* Derby. In Uruguay, Betancor et al. (2010) demonstrated the presence of a *S*. Derby variant in eggs that lacked virulence factors and was impaired in the ability to cause human infection. Unfortunately, these strains were not characterized further than the serovar level. Additional experiments are required to evaluate adhesion, invasion, multiplication and virulence of the various Derby clades before concluding on whether there are differences in their ability to infect humans.

This study demonstrated that the four genomic clusters identified in pork and poultry in France are unequally represented in human clinical strains. Those four genomic clusters were also recovered in other European countries (Hauser et al., 2011; Bonardi, 2017). Most of the human clinical cases studied here were associated to pork. In fact, 94% of human reported cases corresponds to lineages associated to the pork sector (ST39, ST40, and ST682). ST40 contributed to 71% of human cases. Moreover, the main antimicrobial resistance profile [*aadA2*, *sul1* and *tet* (A)], previously identified in the pork sector in Spain (Valdezate et al., 2005) and France (Kerouanton et al., 2013), is associated with a specific ST40 clade. This specific clade with its specific antimicrobial resistance profile seems to have been present in the European pork sector at least over the past two decades.

Nowadays, the power of real-time genomic surveillance of *Salmonella* isolates of both food and clinical origins is undeniable for outbreak investigation. Nonetheless, retrospective analysis of sporadic cases is an underestimated tool to mitigate human salmonellosis by identifying the appropriate interventions to be implemented. Finally, the main output of this study is that intervention measures to prevent human salmonellosis due to *S.* Derby should probably focus on the pork sector in order to be cost effective.

## Data Availability Statement

Genomics sequences used in this project are available online on the NCBI network under the accession number: PRJNA391404 (available at: https://www.ncbi.nlm.nih.gov/bioproject/PRJNA391404). Details for each genomic assembly are summarized in Supplementary Table S1, including the accession codes for each strain.

## Author Contributions

SC-S piloted and administered the project. SC-S and YS designed and developed the experiments. YS and LG carried out the experiments and the analyses. SC-S and M-YM provided acquisitions. YS, SC-S, SG, and M-YM drafted the manuscript. SL and F-XW provided the human clinical strain genomes and epidata, participated in discussions, and reviewed the report.

## Conflict of Interest

The authors declare that the research was conducted in the absence of any commercial or financial relationships that could be construed as a potential conflict of interest.

## Footnotes

1 https://cge.cbs.dtu.dk/services/MLST/

2 https://itol.embl.de/

## Supplementary Material

FIGURE S1 Phylogenetic tree of the French *Salmonella* Derby isolates belonging to ST40. The source of the strainis reported: pork (red), poultry (green), and human (blue). Bootstraps are presented by a color range on the nodes, varying from green (100% of the bootstraps supports the given node) to red (4% of the bootstraps supports the node). Red, purple, and greengroups are underlined in the image. Red group belongs to Cluster ST40 clade 1. Purple and green groups belong to Cluster ST40 clade 2. The predicted antibiotic resistance genes are indicated on the right-hand side. Red cubes correspond to the resistancegenes to aminoglycoside antibiotics, blue circles to beta-lactams, green triangles to sulfonamides, orange circles to trimethoprim, purple triangles to tetracycline, yellow squares to phenicols and light purple squares to quinolones.

FIGURE S2 Production basins of pork sector in France.

TABLE S1 Genome accession and epidemiologic data for the genome collection.

TABLE S2 Antimicrobial susceptibility profiles.

## References

[B1] Achtman M. Wain J. Weill F. X. Nair S. Zhou Z. Sangal V. (2012). Multilocus sequence typing as a replacement for serotyping in *Salmonella enterica*. *PLoS Pathog.* 8:e1002776 10.1371/journal.ppat.1002776 PMC3380943 22737074

[B2] Adam K. Brülisauer F. (2010). The application of food safety interventions in primary production of beef and lamb: a review. *Int. J. Food Microbiol.* 141 S43–S52. 10.1016/j.ijfoodmicro.2009.12.020 20097438

[B3] Andoh L. A. Ahmed S. Olsen J. E. Obiri-Danso K. Newman M. J. Opintan J. A. (2017). Prevalence and characterization of *Salmonella* among humans in Ghana. *Trop. Med. and Health* 45:3 10.1186/s41182-017-0043-z PMC5301370 28194090

[B4] Ao T. T. Feasey N. A. Gordon M. A. Keddy K. H. Angulo F. J. Crump J. A. (2015). Global burden of invasive nontyphoidal *Salmonella* disease, 2010. *Emerg. Infect. Dis.* 21 941–949. 10.3201/eid2106.140999 PMC4451910 25860298

[B5] Baker-Austin C. Wright M. S. Stepanauskas R. McArthur J. V. (2006). Co-selection of antibiotic and metal resistance. *Trends Microbiol.* 14 176–182. 10.1016/j.tim.2006.02.006 16537105

[B6] Betancor L. Pereira M. Martinez A. Giossa G. Fookes M. Flores K. (2010). Prevalence of *Salmonella enterica* in poultry and eggs in Uruguay during an epidemic due to *Salmonella enterica* serovar Enteritidis. *J. Clin. Microbiol.* 48 2413–2423. 10.1128/jcm.02137-09 20484605 PMC2897505

[B7] Beutlich J. Jahn S. Malorny B. Hauser E. Huhn S. Schroeter A. (2011). Antimicrobial resistance and virulence determinants in European *Salmonella* genomic island 1-positive *Salmonella enterica* isolates from different origins. *Appl. Environ. Microbiol.* 77 5655–5664. 10.1128/aem.00425-11 21705546 PMC3165277

[B8] Bonardi S. (2017). *Salmonella* in the pork production chain and its impact on human health in the European Union. *Epidemiol. Infect.* 145 1513–1526. 10.1017/s095026881700036x 28241896 PMC9203350

[B9] Botteldoorn N. Herman L. Rijpens N. Heyndrickx M. (2004). Phenotypic and molecular typing of *Salmonella* strains reveals different contamination sources in two commercial pig slaughterhouses. *Appl. Environ. Microbiol.* 70 5305–5314. 10.1128/AEM.70.9.5305-5314.2004 15345414 PMC520922

[B10] Cai Y. Tao J. Jiao Y. Fei X. Zhou L. Wang Y. (2016). Phenotypic characteristics and genotypic correlation between *Salmonella* isolates from a slaughterhouse and retail markets in Yangzhou, China. *Int. J. Food Microbiol.* 222 56–64. 10.1016/j.ijfoodmicro.2016.01.020 26851738

[B11] CDC (2017). *National Enteric Disease Surveillance: Salmonella Annual Report.* Atlanta, DA: CDC.

[B12] Chaves B. D. Han I. Y. Dawson P. L. Northcutt J. K. (2011). Survival of artificially inoculated *Escherichia coli* and *Salmonella* Typhimurium on the surface of raw poultry products subjected to crust freezing. *Poult. Sci.* 90 2874–2878. 10.3382/ps.2011-01640 22080028

[B13] Cui S. Li J. Sun Z. Hu C. Jin S. Li F. (2009). Characterization of *Salmonella enterica* isolates from infants and toddlers in Wuhan. China. *J. Antimicrob. Chemother*. 63 87–94. 10.1093/jac/dkn452 18984647

[B14] David J. Danan C. Chauvin C. Chazel M. Souillard R. Brisabois A. (2011). Structure of the French farm-to-table surveillance system for *Salmonella*. *Revue Méd. Vétér.* 162 489–500.

[B15] Deng W. Quan Y. Yang S. Guo L. Zhang X. Liu S. (2017). Antibiotic resistance in *Salmonella* from retail foods of animal origin and its association with disinfectant and heavy metal resistance. *Microb. Drug Resist.* 24 782–791. 10.1089/mdr.2017.0127 29039715

[B16] DGAL (2014). “Bilan 2014 des plans de surveillance et de contrôle,” in *Surveillance Sanitaire des Denrées Animales et Végétales en France.* Boulder, CO: DGAI, 69–74.

[B17] DGAL (2015). “Bilan 2015 des plans de surveillance et de contrôle,” in *Surveillance Sanitaire des Denrées Animales et Végétales en France.* Boulder, CO: DGAI, 112–125.

[B18] ECDC. (2018). *The European Union Summary Report on Trends and Sources of Zoonoses, Zoonotic Agents and Food-Borne Outbreaks in 2017.* Parma: EFSA. 10.2903/j.efsa.2018.5500 PMC7009540 32625785

[B19] EFSA (2008). Report of the task force on zoonoses data collection on the analysis of the baseline survey on the prevalence of *Salmonella* in slaughter pigs, in the EU, 2006–2007 - Part A: *Salmonella* prevalence estimates. *EFSA J.* 6:135r 10.2903/j.efsa.2008.135r

[B20] EFSA (2009). Analysis of the baseline survey on the prevalence of *Salmonella* in holdings with breeding pigs in the EU, 2008 - Part A: *Salmonella* prevalence estimates. *EFSA J.* 7:1377 10.2903/j.efsa.2009.1377

[B21] EFSA (2015). The European Union summary report on trends and sources of zoonoses, zoonotic agents and food-borne outbreaks in 2014. *EFSA J.* 13:4329 10.2903/j.efsa.2015.4329 PMC7009540 32625785

[B22] EFSA (2016). The European Union summary report on trends and sources of zoonoses, zoonotic agents and food-borne outbreaks in 2015. *EFSA J.* 14:e04634 10.2903/j.efsa.2016.4634 PMC7009962 32625371

[B23] EFSA (2017). The European Union summary report on trends and sources of zoonoses, zoonotic agents and food−borne outbreaks in 2016. *EFSA J.* 15:e05077 10.2903/j.efsa.2017.5077 PMC7009962 32625371

[B24] Feasey N. A. Dougan G. Kingsley R. A. Heyderman R. S. Gordon M. A. (2012). Invasive non-typhoidal *Salmonella* disease: an emerging and neglected tropical disease in Africa. *Lancet* 379 2489–2499. 10.1016/S0140-6736(11)61752-2 22587967 PMC3402672

[B25] Felten A. Vila Nova M. Durimel K. Guillier L. Mistou M.-Y. Radomski N. (2017). First gene-ontology enrichment analysis based on bacterial coregenome variants: insights into adaptations of *Salmonella* serovars to mammalian- and avian-hosts. *BMC Microbiol.* 17:222 10.1186/s12866-017-1132-1 PMC5706153 29183286

[B26] Fois F. Piras F. Torpdahl M. Mazza R. Consolati S. G. Spanu C. (2017). Occurrence, characterization, and antimicrobial susceptibility of *Salmonella enterica* in slaughtered pigs in sardinia. *J. Food Sci.* 82 969–976. 10.1111/1750-3841.13657 28226178

[B27] Gosling R. J. Mueller-Doblies D. Martelli F. Nunez-Garcia J. Kell N. Rabie A. (2018). Observations on the distribution and persistence of monophasic *Salmonella* Typhimurium on infected pig and cattle farms. *Vet. Microbiol.* 227 90–96. 10.1016/j.vetmic.2018.10.032 30473358

[B28] Grimont P. Weill F.-X. (2007). *Antigenic formulae of the Salmonella serovars. 9th [Online]. Paris: WHO collaborating center for reference and research on Salmonella, Institut Pasteur.* Available online at: https://www.pasteur.fr/sites/default/files/veng_0.pdf (accessed April 6, 2020).

[B29] Hauser E. Hebner F. Tietze E. Helmuth R. Junker E. Prager R. (2011). Diversity of *Salmonella enterica* serovar Derby isolated from pig, pork and humans in Germany. *Int. J. Food Microbiol.* 151 141–149. 10.1016/j.ijfoodmicro.2011.08.020 21917347

[B30] Hayward M. R. Petrovska L. Jansen V. A. Woodward M. J. (2016). Population structure and associated phenotypes of *Salmonella enterica* serovars Derby and Mbandaka overlap with host range. *BMC Microbiol.* 16:15 10.1186/s12866-016-0628-4 PMC4743429 26846255

[B31] Henry A.-E. (2014). *Étude de la Distribution de Salmonella Comme un Descripteur des Enjeux de Biosécurité Dans un Réseau de Production: Elevages et Abattoir de Porcs.* Montreal, QC: Université de Montréal.

[B32] Huang X. Huang Q. Dun Z. Huang W. Wu S. Liang J. (2016). Nontyphoidal *Salmonella* Infection, Guangdong Province, China, 2012. *Emerg. Infect. Dis.* 22 726–729. 10.3201/eid2204.151372 26982074 PMC4806953

[B33] IFIP (2014). *Le Porc par les Chiffres 2014-2015.* Laxenburg: IFIP.

[B34] Kerouanton A. Rose V. Weill F. X. Granier S. A. Denis M. (2013). Genetic diversity and antimicrobial resistance profiles of *Salmonella enterica* serotype Derby isolated from pigs, pork, and humans in France. *Foodborne Pathog. Dis.* 10 977–984. 10.1089/fpd.2013.1537 23944749

[B35] Leclerc V. Moury F. Noel V. Berta-Vanrullen I. Cadel-Six S. Lailler R. (2016). Le réseau *Salmonella*, un dispositif de surveillance des salmonelles sur la chaîne alimentaire: bilan 2015. *Bull. Epidémiol. Santé Anim. Aliment.* 77 75–81.

[B36] Letunic I. Bork P. (2016). Interactive tree of life (iTOL) v3: an online tool for the display and annotation of phylogenetic and other trees. *Nucleic Acids Res.* 44 W242–W245. 10.1093/nar/gkw290 27095192 PMC4987883

[B37] Malpel G. P. Marigeaud M. Marty S. (2014). *La Filière Volaille de Chair, Rapport CGAAER.* Paris: Inspection générale des finances.

[B38] Markowski C. A. Markowski E. P. (1990). Conditions for the Effectiveness of a Preliminary Test of Variance. *Am. Stat.* 44 322–326. 10.2307/2684360

[B39] McKenna A. Hanna M. Banks E. Sivachenko A. Cibulskis K. Kernytsky A. (2010). The genome analysis toolkit: a mapreduce framework for analyzing next-generation DNA sequencing data. *Genome Res.* 20 1297–1303. 10.1101/gr.107524.110 20644199 PMC2928508

[B40] Méheust D. Chevance A. Moulin G. (2016). *Suivi des Ventes de Médicaments Vétérinaires Contenant des Antibiotiques en France en 2015.* Paris: Anses.

[B41] Menard J.-N. Nil A. Pierre T. (2015). *Bilan Diagnostic des Bassins de Production de Volailles de Chair. CGAAER_11044_2012_Rapport_cle0dd2cb.* Paris: République Française.

[B42] Moura A. Soares M. Pereira C. Leitao N. Henriques I. Correia A. (2009). INTEGRALL: a database and search engine for integrons, integrases and gene cassettes. *Bioinform* 25 1096–1098. 10.1093/bioinformatics/btp105 19228805

[B43] Mughini-Gras L. Kooh P. Augustin J.-C. David J. Fravalo P. Guillier L. (2018). Source attribution of foodborne diseases: potentialities, hurdles, and future expectations. *Front. Microbiol.* 9:1983 10.3389/fmicb.2018.01983 PMC6129602 30233509

[B44] Mughini-Gras L. Kooh P. Fravalo P. Augustin J. C. Guillier L. David J. (2019). Critical orientation in the jungle of currently available methods and types of data for source attribution of foodborne diseases. *Front. Microbiol.* 10:2578 10.3389/fmicb.2019.02578 PMC6861836 31798549

[B45] Mushin R. (1948). An outbreak of gastro-enteritis due to *Salmonella* Derby. *J. Hyg.* 46 151–157. 10.1017/S0022172400036238 18882227 PMC2235082

[B46] Newell D. G. Koopmans M. Verhoef L. Duizer E. Aidara-Kane A. Sprong H. (2010). Food-borne diseases - the challenges of 20 years ago still persist while new ones continue to emerge. *Int. J. Food Microbiol.* 139(Suppl. 1) S3–S15. 10.1016/j.ijfoodmicro.2010.01.021 20153070 PMC7132498

[B47] Pal C. Bengtsson-Palme J. Kristiansson E. Larsson D. G. J. (2015). Co-occurrence of resistance genes to antibiotics, biocides and metals reveals novel insights into their co-selection potential. *BMC Genomics* 16:964 10.1186/s12864-015-2153-5 PMC4650350 26576951

[B48] Petrovska L. Mather A. E. AbuOun M. Branchu P. Harris S. R. Connor T. (2016). Microevolution of monophasic *Salmonella* typhimurium during epidemic. United Kingdom, 2005–2010. *Emerg. Infect. Dis.* 22 617–624. 10.3201/eid2204.150531 26982594 PMC4806966

[B49] Ran L. Wu S. Gao Y. Zhang X. Feng Z. Wang Z. (2011). Laboratory-based surveillance of nontyphoidal *Salmonella* infections in China. *Foodborne Pathog. Dis.* 8 921–927. 10.1089/fpd.2010.0827 21492026

[B50] Rehman M. A. Yin X. Persaud-Lachhman M. G. Diarra M. S. (2017). First detection of a fosfomycin resistance gene, fosA7, in *Salmonella enterica* serovar heidelberg isolated from broiler chickens. *Antimicrob. Agents Chemother.* 61 410–417. 10.1128/aac.00410-17 PMC5527569 28533247

[B51] Rostagno M. H. Hurd H. S. McKean J. D. Ziemer C. J. Gailey J. K. Leite R. C. (2003). Preslaughter holding environment in pork plants is highly contaminated with *Salmonella enterica*. *Appl. Environ. Microbiol.* 69 4489–4494. 10.1128/aem.69.8.4489-4494.2003 12902233 PMC169143

[B52] Royston P. (1995). Remark AS R94: a remark on algorithm AS 181: the w-test for normality. *J. R. Stat. Soc. Series C (Appl. Statist.)* 44 547–551. 10.2307/2986146

[B53] Rubbo S. D. (1948). Cross-infection in hospital due to *Salmonella* Derby. *J. Hyg.* 46 158–163. 10.1017/S002217240003624X 18882228 PMC2235095

[B54] Ruiz M. Rodriguez J. C. Escribano I. Royo G. (2004). Available options in the management of non-typhi *Salmonella*. *Expert Opin. Pharmacother* 5 1737–1743. 10.1517/14656566.5.8.1737 15264988

[B55] Sanders E. Sweeney F. J. Jr. Friedman E. A. Boring J. R. Randall E. L. (1963). An outbreak of hospital-associated infections due to *Salmonella* Derby. *JAMA* 186 984–986. 10.1001/jama.1963.03710110036007 14066720

[B56] Schmidt H. A. Minh B. Q. von Haeseler A. Nguyen L.-T. (2014). IQ-TREE: a fast and effective stochastic algorithm for estimating maximum-likelihood phylogenies. *Mol. Biol. Evol.* 32 268–274. 10.1093/molbev/msu300 25371430 PMC4271533

[B57] Schmidt J. W. Brichta-Harhay D. M. Kalchayanand N. Bosilevac J. M. Shackelford S. D. Wheeler T. L. (2012). Prevalence, Enumeration, Serotypes, and Antimicrobial Resistance Phenotypes of <span class=”named-content genus-species” id=”named-content-1”>*Salmonella enterica*</span> Isolates from Carcasses at Two Large United States Pork Processing Plants. *Appl. Environ. Microb.* 78 2716–2726. 10.1128/aem.07015-11 PMC3318825 22327585

[B58] Sévellec Y. Felten A. Radomski N. Granier S. A. Le Hello S. Petrovska L. (2019). Genetic diversity of *Salmonella* derby from the poultry sector in Europe. *Pathoges* 8:46 10.3390/pathogens8020046 PMC6630433 30987404

[B59] Sévellec Y. Granier S. A. Radomski N. Felten A. Le Hello S. Feurer C. (2018a). Complete genome sequence of *Salmonella enterica* subsp. *enterica* serotype derby, associated with the pork sector in France. *Microbiol. Resour. Announc.* 7 1027–1018. 10.1128/MRA.01027-18 PMC6256686 30533663

[B60] Sévellec Y. Vignaud M. L. Granier S. A. Lailler R. Feurer C. Le Hello S. (2018b). Polyphyletic nature of *Salmonella enterica* serotype derby and lineage-specific host-association revealed by genome-wide analysis. *Front. Microbiol.* 9:891 10.3389/fmicb.2018.00891 PMC5966662 29867804

[B61] Simon S. Trost E. Bender J. Fuchs S. Malorny B. Rabsch W. (2017). Evaluation of WGS based approaches for investigating a food-borne outbreak caused by *Salmonella enterica* serovar Derby in Germany. *Food Microbiol.* 71 46–54. 10.1016/j.fm.2017.08.017 29366468

[B62] Sofos J. N. (2008). Challenges to meat safety in the 21st century. *Meat Sci.* 78 3–13. 10.1016/j.meatsci.2007.07.027 22062090

[B63] Stamatakis A. (2014). RAxML version 8: a tool for phylogenetic analysis and post-analysis of large phylogenies. *Bioinform.* 30 1312–1313. 10.1093/bioinformatics/btu033 PMC3998144 24451623

[B64] Ung A. Baidjoe A. Y. Van Cauteren D. Fawal N. Fabre L. Guerrisi C. (2019). Disentangling a complex nationwide *Salmonella* Dublin outbreak associated with raw-milk cheese consumption, France, 2015 to 2016. *Euro. Surveill.* 24:1700703 10.2807/1560-7917.Es.2019.24.3.1700703 PMC6344836 30670140

[B65] Valdezate S. Vidal A. Herrera-Leon S. Pozo J. Rubio P. Usera M. A. (2005). *Salmonella* Derby clonal spread from pork. *Emerg. Infect. Dis.* 11 694–698. 10.3201/eid1105.041042 15890121 PMC3320352

[B66] Weill F. Le Hello S. (2014). *Bilan des Activités 2013 du Centre National de Référence des Esherichia coli, Shigella et Salmonella [Online]. Centre National de Référence des Escherichia coli, Shigella et Salmonella.* Available online at: https://www.pasteur.fr/fr/sante-publique/CNR/les-cnr/escherichia-coli-shigella-salmonella/rapports-d-activite (accessed December 12, 2016).

[B67] Weill F.-X. Le Hello S. Lefevre S. Renaudat C. Bonacorsi S. Mariani-Kurkdjian P. (2018). “Rapport d’activité annuel 2018 Centre National de Référencedes *Escherichia coli, Shigella et Salmonella*,” in *Unité de Recherche et d’Expertise des Bactéries Pathogènes Entériques*, ed. Pasteur I. (Paris: Institut Pasteur).

[B68] WHO (2018). *Salmonella (non-typhoidal) - Fact sheet [Online].* Available online at: http://www.who.int/mediacentre/factsheets/fs139/en/ (Accessed Febraury 20, 2018).

[B69] Zankari E. Hasman H. Cosentino S. Vestergaard M. Rasmussen S. Lund O. (2012). Identification of acquired antimicrobial resistance genes. *J. Antimicrob. Chemother.* 67 2640–2644. 10.1093/jac/dks261 22782487 PMC3468078

[B70] Zheng H. Hu Y. Li Q. Tao J. Cai Y. Wang Y. (2017). Subtyping *Salmonella enterica* serovar Derby with multilocus sequence typing (MLST) and clustered regularly interspaced short palindromic repeats (CRISPRs). *Food Control* 73(Part B) 474–484. 10.1016/j.foodcont.2016.08.051

