# Supplementary figures and images for: Source Attribution Study of Sporadic Salmonella Derby Cases in France
Source: Front Microbiol. 2020 May 14;11:889. doi: 10.3389/fmicb.2020.00889 (PMC7240076; doi:10.3389/fmicb.2020.00889)

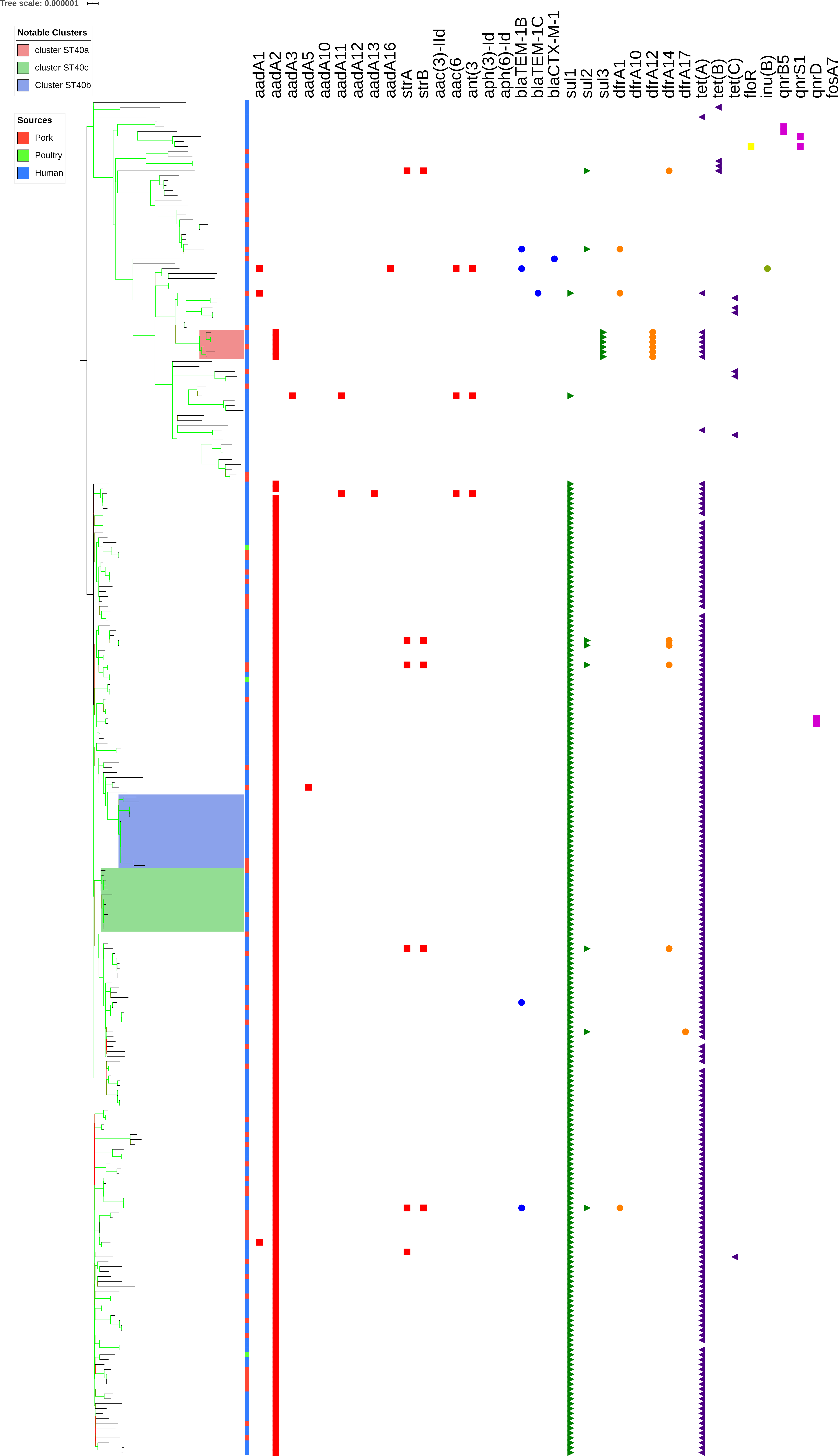

Supplement: FIGURE S1 — Phylogenetic tree of the French Salmonella Derby isolates belonging to ST40. The source of the strainis reported: pork (red), poultry (green), and human (blue). Bootstraps are presented by a color range on the nodes, varying from green (100% of the bootstraps supports the given node) to red (4% of the bootstraps supports the node). Red, purple, and greengroups are underlined in the image. Red group belongs to Cluster ST40 clade 1. Purple and green groups belong to Cluster ST40 clade 2. The predicted antibiotic resistance genes are indicated on the right-hand side. Red cubes correspond to the resistancegenes to aminoglycoside antibiotics, blue circles to beta-lactams, green triangles to sulfonamides, orange circles to trimethoprim, purple triangles to tetracycline, yellow squares to phenicols and light purple squares to quinolones. [file Image_1.png]

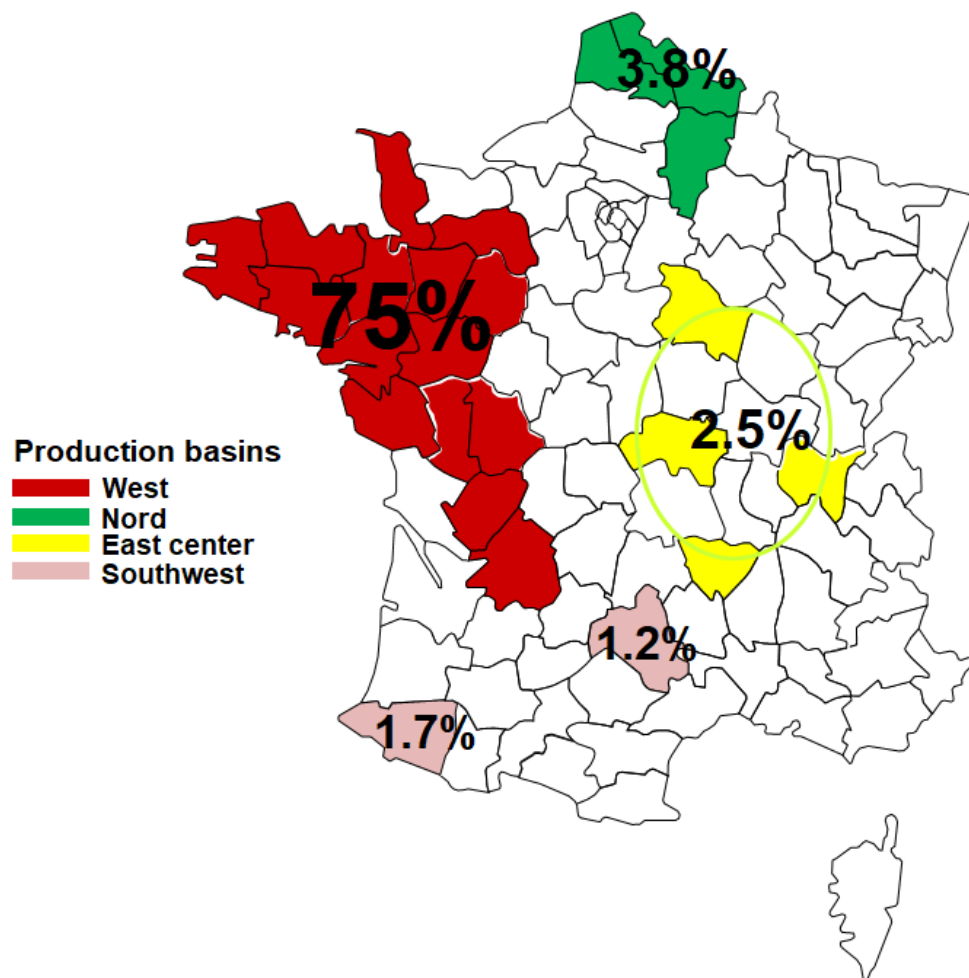

Supplementary figure 2. Production basins of pork sector in France

Supplement: FIGURE S2 — Production basins of pork sector in France. [file Image_2.pdf]
